# Medical students’ reflective capacity and its role in their critical thinking disposition

**DOI:** 10.1186/s12909-023-04163-x

**Published:** 2023-03-30

**Authors:** Zohreh Khoshgoftar, Maasoumeh Barkhordari-Sharifabad

**Affiliations:** 1 Virtual School of Medical Education and Management, Shahid Beheshti University of Medical Sciences, Tehran, Iran; 2 Virtual School of Medical Education and Management, Shahid Beheshti University of Medical Sciences, Tehran, Iran; 3 School of Medical Sciences, Yazd Branch, Islamic Azad University, Yazd, Iran

**Keywords:** Reflection, Reflective capacity, Critical thinking, Students, Medical education

## Abstract

**Background:**

Developing reflective capacity and critical thinking is one of the prerequisites of education in health professions, especially medicine. This study aimed to determine the reflective capacity of medical students and its role in their critical thinking disposition.

**Methods:**

In this cross-sectional descriptive research, conducted in 2022, a total of 240 medical intern students were selected using the convenient sampling method. Data were collected using a reflective capacity questionnaire and critical thinking disposition questionnaire and analyzed with descriptive and inferential statistics using SPSS20.

**Results:**

The mean reflective capacity was 4.53 ± 0.50, and mean critical thinking disposition was 127.52 ± 10.85. Among the dimensions of reflection, “active self-appraisal (SA)” and “reflective with others (RO)” had the highest and lowest means, respectively. The dimensions of critical thinking disposition with the highest and lowest means were related to innovation and intellectual maturity, respectively. Reflective capacity and its dimensions were found to have a direct and statistically significant relationship with critical thinking disposition and its dimensions. Regression analysis results showed that reflective capacity accounts for 28% of students’ critical thinking disposition.

**Conclusion:**

The relationship between students’ reflective capacity and their critical thinking disposition has rendered reflection as one of the necessary components of medical education. Thus, determining the learning activities by considering the reflection process and models will be very effective in creating and strengthening critical thinking disposition.

## Background

Thinking and reflecting on what has been learned have been emphasized as key factors in the learning process, one considered a metacognitive phenomenon that can occur before, during, and after the learning process [1, 2]. Reflective capacity is considered one of the important competencies in any healthcare system [3] and refers to “the ability, willingness, and tendency of students to participate in reflective thinking during the course of study and clinical practices” [4]. Reflective capacity is rethinking about one’s own and others’ experiences to make decisions about future behaviors [3, 5]. This capacity is an important ability that allows physicians to be alert, interested, aware, and ready to identify and correct errors [6].

In health professions, it is necessary to cultivate learners who, in addition to clinical abilities, have the ability for problem-solving, clinical reasoning, and self-regulated learning, followed by life-long learning. Reflection is one of the processes that provide the possibility of cultivating and creating such capacities in the learners of health professions [7]. Fostering reflection improves professionalism, which is one of the core competencies of medicine [5, 8]. A review of the relevant literature showed that the reflection process is useful in achieving a higher level in professional life [3, 9, 10]. Reflecting on past experiences leads to deeper learning and better performance [11]. Reflection leads to “transformational learning” and learners gain an understanding of their responsibility in providing health care [4]; moreover, it promotes self-awareness, clinical insight, and quality of care [12, 13], stress management and teamwork [12, 14], and empathy and professionalism [15], and it helps clinicians make difficult or ethical decisions when faced with complex cases in clinical practice [16].

Certainly, the ability to rethink or reflect should be learned and encouraged, indicating that it does not emerge automatically; rather, it requires active effort and energy [17]. Therefore, reflection can be seen as an important learning tool in university education, which leads to the cultivation of students by health professions who, in addition to clinical capabilities, have the ability to solve problems and think critically [7].

The reflective practice, which is an ad hoc process of evaluating and strengthening skills and information acquired or being acquired, is closely related to critical thinking [18]. Critical thinking is one of the main thinking skills. It is a mental process that leads to the purposeful performance of people and prevents the repetition of usual patterns and stereotypes; it further evaluates prejudices, assumptions, and types of information and discusses different aspects, meanings, and results [19]. Reflection has been mentioned as one of the processes that lead to the improvement of critical thinking ability [20]. In other words, incorporating reflection into practice can support the development of critical thinking skills and the professional progression of students [21]. Educators can encourage the development of critical thinking in students using reflection methods, which allow students to understand how their behaviors and attitudes are perceived by others [22]. Of course, to improve critical thinking, affective dispositions in addition to basic cognitive abilities should be considered. A person’s critical thinking disposition refers to certain features of their thinking process that cultivate critical thinking [23].

Based on current knowledge, reflective practice is important in gaining professional expertise, and experience alone is not enough. Hence, it is essential to teach reflection as a necessary ability for students of health professions to strengthen it to create meaningful and continuous learning and develop professional performance. This study determined the reflective capacity of medical students and investigated its role in critical thinking disposition as both of these abilities are emphasized in the medical curriculum.

## Methods

### Study Design

This descriptive-cross-sectional study was conducted in 2022. The research community consisted of all students studying in the medical schools of medical sciences universities of Iran. Samples size was estimated to be 240 using the sample size formula, with a confidence level of less than 5%, test power of 80% according to a previous similar study [24], and an approximate correlation coefficient of at least r = 0.18. Sampling was done by the convenient sampling method. Inclusion criteria comprised being employed, studying at the internship level, and willingness to participate in the study. Anyone lacking interest in participating in the study or failing to complete the scales was excluded.

## Data Collection Tools

The following tools were used to collect data in the current research.

1. ***Demographic Information Questionnaire***: This questionnaire gathered participant demographic information including age, gender, grand point average (GPA), and marital status.
2. **Reflective Capacity Scale**: Developed by Priddis and Rogers in 2018 [25], this scale contains 16 items that measure the four dimensions of Reflective-in-action (RiA), Reflective-on-action (RoA), Reflective with others (RO), and Self-appraisal (SA) [4, 25]. These sub-components together form the scale of reflective capacity. Items 4, 7, 11, and 14 are related to the dimension of RiA, items 2, 8, 10, and 13 are related to the dimension of RoA, items 1, 5, 12, and 16 are related to the dimension of RO, and items 3, 6, 9 and 15 are related to the dimension of SA. All items in the scale are scored based on a 6-point Likert scale (from “not at all” to “very much”), and grades range from 1 to 6 with higher scores indicating a greater capacity for reflection. The current study used this scale for the first time in Iran; therefore, after obtaining permission from the original developer of the scale, it was translated by two translators using the forward-backward method. After the translation was approved, its face and content validities were checked. Face validity was examined qualitatively by asking the opinion of ten medical intern students about its difficulty and ambiguity. To check the content validity of the scale, it was given to ten experts in the field of medical education and reflection, and they were asked to give their professional subjective judgment and viewpoints on the relevance, necessity, representativeness, and comprehensiveness of the items. Construct validity was surveyed with exploratory and confirmatory factor analysis using the questionnaires completed by 320 medical students, who were selected using convenience sampling. Exploratory factor analysis identified four factors, which accounted for 63.79% of the variance in the scores. In the confirmatory factor analysis, the values of the fit indices confirmed the appropriate fit of the model. To establish the reliability of the tool, the questionnaire was given to 20 random samples from the studied population. Cronbach’s α coefficient was 0.83 for the overall scale.
3. ***Critical Thinking Disposition Scale***: Developed by Ricketts (2003) [26], this scale contains 33 items in the 3 dimensions of innovation, intellectual maturity, and mental engagement. Eleven items (1, 5, 7, 11, 14, 17, 24, 25, 26, 28, and 29) relate to innovation; 9 items incluidng items (2, 12, 15, 19, 23, 30, 31, 32 and 33) relate to intellectual maturity; and the remaining 13 items are related to mental engagement. This scale is graded using a 5-point Likert scale from completely disagree (1 point) to completely agree (5 points). Items 2, 12, 15, 19, 23, 30, 32, and 33 are graded in reverse, so that “I completely agree” is given 1 point and “I completely disagree” is given 5 points. The total score of critical thinking disposition is obtained by summing up of the three subscale scores; based on the mean total score, strong, medium and weak disposition may be determined. A total score of 135.31 + describes a strong disposition, a score of 108.91 to 135.30 indicates a mean disposition, and a score of 108.90 or less indicates a weak disposition to critical thinking. The Persian version of the scale was culturally and linguistically adapted by Pakmehr et al. [27] and has been used in several studies to evaluate critical thinking dispositions of Iranian medical, nursing, and midwifery students [28, 29]. To verify the validity of the Persian version of this scale, its face and content validity was confirmed by 6 professors of educational sciences [30]. The results of construct validity determined using confirmatory factor analysis indicated the appropriate fit of the model [27]. The overall alpha was 0.74, and the subscales alphas ranged from 0.71 to 0.78 [30]. In this study, Cronbach’s α coefficient was 0.78.

### Data Analysis

After coding, the data was imported into SPSS20 and analyzed using descriptive statistics (frequency distribution tables, calculation of numerical indices) and inferential statistics (independent t-tests, Pearson’s correlation coefficient, and linear regression) (*p* < 0.05). Before running the tests, the normal distribution of the data was confirmed using the Kolmogorov-Smironov (KS) test (*p* > 0.05).

## Findings

In total, 240 questionnaires were completed and analyzed. Participants had a mean age of 24.75 ± 1.77 years and mean GPA of 16.73 ± 1.13. The majority of participants were female (52.5%) and single (87.9%) (Table 1).

**Table 1:** Demographic Information of Participants

Variable		Mean ± SD	Frequency (%)
Age		24.75 ± 1.77	
Grand point average (GPA)		16.73 ± 1.13	
Gender	Female		126 (52.5)
	Male		114 (47.5)
Marital status	Married		129 (12.1)
	Single		211 (87.9)

As displayed in Table 2, the mean reflective capacity was 4.53 ± 0.50 and that of critical thinking disposition was 127.52 ± 10.85. Among the dimensions of reflection, SA and RO had the highest and lowest means, respectively. To calculate the weight of each dimension in the critical thinking disposition score, all scores were converted into a coefficient of 100. The highest mean was related to innovation and the lowest mean was related to intellectual maturity (Table 2).

The results indicated that critical thinking disposition was at an average level in 164 (68.3%), at a low level in 13 (5.4%), and at a high level in 63 (26.3%) participants.

**Table 2:** Descriptive findings of reflective capacity, and critical thinking disposition

Variables	Number of items	Min.-max.	Mean ± SD.	Standard Score (Mean ± SD.) of 100
Reflective-in-action (RiA)	4	2.25-6.00	4.42 ± 0.68	-
Reflective-on-action (RoA)	4	3.00–6.00	4.56 ± 0.64	-
Reflective with others (RO)	4	2.25–5.75	4.36 ± 0.62	-
Self-appraisal (SA)	4	2.50-6.00	4.78 ± 0.65	-
**Reflective capacity**	16	2.88–5.81	4.53 ± 0.50	-
Innovation	11	35–54	44.88 ± 4.06	77.00 ± 9.24
Intellectual maturity	9	21–37	30.62 ± 3.21	60.05 ± 8.92
Mental engagement	13	33–65	52.02 ± 6.55	75.04 ± 12.61
**Critical thinking disposition**	33	103–152	127.52 ± 10.85	71.60 ± 8.22

Pearson’s correlation test revealed a direct and significant statistical relationship between “reflective capacity and its dimensions” and “critical thinking disposittion and its dimensions” (Table 3).

**Table 3:** Correlation matrix of reflective capacity, and critical thinking disposition

Variables	1	2	3	4	5	6	7	8	9
1- Reflective-in-action (RiA)	1								
2- Reflective-on-action (RoA)	0.57^**^	1							
3- Reflective with others (RO)	0.35^**^	0.40^**^	1						
4- Self-appraisal (SA)	0.51^**^	0.64^**^	0.19^**^	1					
5- Reflectiva capacity	0.80^**^	0.85^**^	0.62^**^	0.77^**^	1				
6- Innovation	0.26^**^	0.34^**^	0.29^*^	0.39^**^	0.44^**^	1			
7- Intellectual maturity	0.27^**^	0.28^**^	0.31^**^	0.14^*^	0.33^**^	0.17^**^	1		
8- Mental engagement	0.28^**^	0.43^**^	0.26^**^	0.43^**^	0.46^**^	0.78^**^	0.04	1	
9- Critical thinking disposition	0.34^**^	0.49^**^	0.36^**^	0.45^***^	0.43^**^	0.89^**^	0.39^**^	0.91^**^	1

** Correlation is significant at the 0.01 level (2-tailed).

* Correlation is significant at the 0.05 level (2-tailed).

Based on linear regression using the enter method, the coefficient of correlation (R) between critical thinking disposition as the dependent variable and reflective capacity as the predictor variable was 0.53 and the square of the coefficient (R^2^) was 0.28. The results of analysis of variance (F [1, 238] = 95.38, *p* < 0.001) showed that this amount of R^2^ is significant (Table 4).

**Table 4:** Linear regression analysis for critical thinking disposition

Predictor	B	Standardized beta	T	P
(Constant)	74.88		13.80	< 0.001
Reflective capacity	0.72	0.53	9.76	< 0.001

F [1, 238] = 95.38; *p* < 0.001; R = 0.53; adjusted R^2^ = 0.28.

Based on the results of Pearson’s correlation coefficient test, neither age nor GPA had a statistically significant relationship with either reflective capacity or critical thinking disposition (*p* > 0.05).﻿ Moreover, the independent t-test showed that there was no statistically significant difference between reflective capacity and gender (*p* = 0.173); yet, the difference between reflective capacity and critical thinking disposition was significant (*p* = 0.003); the mean score of critical thinking disposition in men (129.72 ± 10.21) was significantly higher than that of women (125.53 ± 11.06).

The independent t-test revealed a statistically significant difference between reflective capacity and critical thinking disposition in terms of marital status (*p* < 0.001). Reflective capacity was significantly higher in single people (4.58 ± 0.47) compared to married people (4.17 ± 0.51). Additionally, single people with a mean score of 128.72 ± 10.27 had a significantly higher critical thinking disposition compared to married people with a mean score of 118.79 ± 11.12.

## Discussion

This research was conducted to determine the reflective capacity and its role in critical thinking disposition of medical students. It is the first study in Iran to have used the reflective capacity scale to examine the reflective capacity of Iranian medical students.

Reflective capacity is one of the important competencies in the health care system [3]. The results of the present study showed that the amount of reflective capacity of medical students (4.53 ± 0.50) was at a medium to high level. Consistent with our results, Rogers et al. (2019) reported the mean score of 4.16 ± 0.53 for reflective capacity among medical students in a university in Colorado, U.S.A. This mean was 4.27 ± 0.68 in mental health professionals and 3.51 ± 1.02 in the general population [4]. Gustafsson et al. (2021) conducted their study on nurses enrolled in advanced level specialized training at two universities in northern Sweden and showed that the mean score of reflective capacity was 4.19 [31]. The results of a study on students in Spain indicated that students’ reflective capacity was at an almost average level (3.88) [32]. As can be seen, the mean scores of reflective capacity are lower in these studies than in the current one. Nonetheless, another study that examined reflective capacity in Scottish students trained in three different initial teacher education programs reported an approximate mean of 4.85 [33], which is slightly higher than in the present study.

It has been largely proven that reflection is not a spontaneous process but one that can be controlled and taught. Reflective capacity can be cultivated to the point where it even becomes a habit. Hence, the reason for the difference in research results can be attributed to the community, the research environment, and different teaching methods and techniques in universities. Educators play an important role in creating reflective capacity by providing a learning environment that facilitates real reflection. They can strengthen the capacity of reflection in learners by designing and using valid and structured methods during clinical practice.

Reflective capacity comprises four dimensions: RiA, RoA, RO, and SA. RiA involves considering prior beliefs, thoughts, and feelings of the individual and the client during the interaction that can influence the interaction. RoA is related to the interaction with the client and reflecting on what was said and done. RO includes things like gaining new awareness, perspectives, and insights while examining interaction and performance processes with others. Finally, SA consists of thinking about strengths and weaknesses when working with clients, improving abilities, and critically evaluating strategies and techniques used when working with clients [25]. In the present study, the dimension of SA had the highest mean (4.78 ± 0.65) and the dimension of RO had the lowest mean (4.36 ± 0.62). These results were contrary to the research findings of Khalil and Hashish (2022), who reported RO and SA dimensions had the highest and lowest averages, respectively [23]. Participants in the study by Priddis and Rogers (2018) obtained the highest mean in the dimension of RO (4.69 ± 0.68) and the lowest mean in the dimension of RiA (4.31 ± 0.70); the mean SA score in their study was 4.46 ± 0.74 [25]. In the study by Gustafsson et al. (2021), the lowest mean was related to RiA (3.879) [31]. RO having the lowest mean in the current study may be attributed to culture. Iranians have learned the ways of individual solutions to reach basic goals and values more than any other nation [34].

The results of the present study showed that the mean critical thinking disposition was 127.52 ± 10.85 and the majority of students had an average level of critical thinking disposition. The highest mean pertained to innovation, and the lowest mean was related to intellectual maturity. A study conducted on medical students in China suggested that almost 60% of the students had a positive critical thinking disposition; the highest mean belonged to the “searching dimension”, while the lowest mean was related to the truth-seeking dimension [35]. Of course, the California Critical Thinking Disposition Questionnaire was used in the mentioned study, the dimensions of which were different from the questionnaire used in the present study. Nonetheless, Bixler et al. demonstrated that medical students at the School of Medicine, Ohio State University, had relatively strong critical thinking tendencies [36]. Moreover, in a study conducted on medical students of Gonabad/Iran, the mean score of critical thinking disposition (143.57 ± 23.59) [37] was higher than in the current study; unlike the present study, the highest mean was related to commitment and the lowest mean pertained to innovation [37]. The mean score of critical thinking disposition in medical students of Jundishapour/Iran was 70.75 ± 11.12, and the highest and lowest means, respectively, were related to the dimension of mental engagement and innovation [38]; this is inconsistent with the results of the present study. This disparity can be attributed to differences in research communities, education, and culture. The results of the present study indicate that Iranian medical education needs to be reformed in order to develop critical thinking disposition in students. The use of active teaching methods such as case studies [39], learning based on problem solving [40], and concept maps [41] by medical educators can help improve students’critical thinking dispositions and skills.

According to Ricketts (2017), innovation shows one’s background to search for truth. A person’s intellectual maturity shows that they are aware of the complexity of real issues and know that there may be more than one solution to a problem. Mental engagement discloses how much a person is looking for opportunities to reason [42]. Considering that intellectual maturity in this research has been assigned the lowest score, and considering the complex nature of the issues in medicine, it seems that the Iranian medical schools should pay more attention to this dimension in medical education, a point that should be taken as a serious warning.

The currents findings revealed statistically significant relationship between reflective capacity and critical thinking disposition, and higher reflection ability includes greater critical thinking disposition. Reflective capacity is a predictor factor of critical thinking disposition. A study conducted on 93 nursing students in Saudi Arabia also showed that reflection performance has a positive and significant correlation with critical thinking disposition. It also has the ability to predict the variance of critical thinking disposition [23]. The results of another study on nurses in Taiwan indicated that reflection significantly affects critical thinking [43]. Hashim (2019) also states that there is a close connection between reflective practice, which is an ad hoc process of evaluating and reinforcing skills and information acquired or in the process of being acquired, with critical thinking. He states that critical thinking and reflective practice are two intertwined processes that play a role in guiding a student [18]. The effect of reflection on critical thinking has also been reported in interventional studies [44–46], and reflection has been introduced as one of the processes that can improve critical thinking [20].

The current findings have important practical implications for current educational programs that aim to improve students’ critical thinking by enhancing their reflective capacity. Our findings that reflective capacity plays a significant role in critical thinking highlight the need for more emphasis on enhancing reflective thinking. Providing reflection exercises, choosing and determining appropriate educational methods [47], deciding on the use of a structured or unstructured approach, creating a learning environment that stimulates and encourages reflection for students [48] can help to create and strengthen reflective capacity and then promote critical thinking.

## Strengths and limitations of the study

Among the strengths of the current study is the first-time use of a reliable and valid assessment instrument to measure the reflective capacity of Iranian medical students. In addition, an adequate sample (N = 240) and 100% overall response rate provided reliable estimates in the study. the present study was limited by the self-report nature of the questionnaires, which created the discussion of social desirability, and some participants may have refused to provide real answers. However, efforts were made to control this confounding variable to a large extent by assuring the participants of the confidentiality of information and the anonymity of the questionnaires. Furthermore, this research was conducted cross-sectionally, which makes it difficult to draw conclusions about causality. The all number of retrieved studies that had used the reflective capacity scale also limited any comparison of the results.

## Conclusion

There was a significant correlation between reflective capacity and critical thinking disposition. In addition, 28% of the variance of the dependent variable (critical thinking disposition) can be predicted by the reflective capacity. The results makes reflection one of the necessary components of medical education. Therefore, determining the learning activities by considering the reflection process and models will be very effective in creating and enhancing critical thinking disposition. Based on the findings of the present study, educators should use effective educational strategies to strengthen students’ reflective capacity, so that the critical thinking disposition increases. It is important to train instructors to learn and apply effective teaching and learning methods in creating students’ reflective capacity.

## Data Availability

The datasets generated and analyzed during the current study are not publicly available due to an agreement with the participants on the confidentiality of the data but are available from the corresponding author on reasonable request.

## List of abbreviations

RiA: Reflective-in-action
RoA: Reflective-on-action
RO: Reflective with others
SA: Self-appraisal
SD: Standard Deviation
KS: Kolmogorov-Smirnov
GPA: Grand point average.
